# A porcine gene, PBK, differentially expressed in the longissimus muscle from Meishan and Large White pig

**DOI:** 10.1590/S1415-47572009000400017

**Published:** 2009-12-01

**Authors:** Liu Yonggang, Zhao Sumei, Pan Hongbin, Gao Shizheng

**Affiliations:** College of Animal Science and Technology, Yunnan Agricultural University, KunmingChina

**Keywords:** pig, PBK, mRNA differential display, RACE

## Abstract

An investigation of differences in gene expression in the longissimus muscle of Meishan and Large White pigs was undertaken, using the mRNA display technique. A fragment of one differentially expressed gene was isolated and sequenced, whereupon the complete cDNA sequence was then obtained by using the rapid amplification of cDNA ends (RACE). The nucleotide sequence of the gene is not related to any known porcine gene. Sequence analysis revealed that the open reading frame of this gene encodes a protein with 322 amino acids, thus displaying high sequence identity with the PDZ binding kinase (PBK) of eleven other animal species – dog, horse, cattle, human, chimpanzee, crab-eating macaque, rhesus monkey, rat, mouse, gray short-tailed opossum and platypus, so it can be defined as the porcine PBK gene. This gene was finally assigned GeneID:100141310. Phylogenetic tree analysis revealed that the swine PBK gene has a closer genetic relationship with the PBK gene of platypus. Gene expression analysis of eight tissues of a Meishan x Large White cross showed that the porcine PBK gene is differentially expressed in various tissues. Our experiment established the primary foundation for further research on this gene.

## Introduction

The mRNA differential display, first described by Liang and Pardee (1992), is a quick and efficient method for identifying and characterizing altered gene expression in different cell types. It has been statistically shown that 80 to120 primer combinations would be sufficient to cover all the transcript populations in the cell (Liang *et al.*, 1993). Furthermore, this technique possesses the following advantages when compared to similar ones, not only as it is based on simple and established methods, but also more than two samples can be compared simultaneously and only a small amount of starting material is needed (Yamazaki and Saito, 2002).

Chinese indigenous pig breeds, such as Meishan, Erhualian and Tongcheng, often possess valuable traits, such as resistance to diseases, high fertility, good maternal qualities, unique product attributes, longevity and the ability to adapt to harsh conditions. European pig breeds, such as the Large White, Landrace and Duroc, are noted for high growth-rate and lean meat content (Pan *et al.*, 2003). Phenotypic variance is mainly determined by genetic differences. The detection of differences in gene expression between Chinese indigenous and European breeds, or finding the differentially expressed genes between the two types, may serve as a basis for understanding the molecular mechanisms of these phenotypic differences.

The present study applied the mRNA differential display technique to the identification of differentially expressed genes in the longissimus muscle from one Chinese indigenous pig breed (Meishan) and one European (Large White).

## Materials and Methods

###  Sample collection, RNA isolation and first-strand cDNA synthesis

Two purebred populations of Large White and Meishan pigs were established in 2007. Longissimus muscle samples were collected from 120-day-old Large White (4 males and 4 females) and Meishan (4 males and 4 females) pigs for mRNA differential display and semi-quantitative RT-PCR identification. Tissues from the spleen, small intestine, heart, liver, lungs, muscles, fat and kidneys were collected from one adult Meishan x Large White cross for later tissue expression profile analysis. The tissues were immediately frozen in liquid nitrogen and stored at -80 °C. Total RNA was extracted from these using the Total RNA Extraction Kit (Gibco, Grand Island, New York, USA). DNase I treatment of total RNA was performed prior to first-strand cDNA synthesis, the latter then being undertaken by RNA reverse transcription, as previously described (Liu *et al.*, 2004).

###  Differential display

Differential display PCR amplification of each reverse transcription product was carried out with ten arbitrary and nine oligo (dT) primers, also according to Liu *et al.* (2004).

The PCR products were then separated on an 8% non-denaturing polyacrylamide gel and displayed by using silver staining (Liu *et al.*, 2004). The differentially expressed gene band was extracted from the gel and used as a template for re-amplification, which, in turn, was carried out with the corresponding oligo(dT) primer and the arbitrary primers used in mRNA differential display (Liu *et al.*, 2004, 2005). The re-amplification products were then cloned into PMD18-T vector (TaKaRa, Dalian, China), to be bi-directionally sequenced according to the commercial fluorometric method. At least five independent clones were sequenced for each PCR product.

###  Semi-quantitative RT-PCR

Semi-quantitative RT-PCR was performed for porcine PBK gene identification and expression profile analysis, as previously described (Liu and Xiong, 2007). In order to eliminate the effect of cDNA concentration, we repeated the RT-PCR five times using 100, 200, 300, 400 and 500 ng of cDNA as templates, respectively. The housekeeping gene, beta-actin (DQ845171), was selected as internal control. The primers used were: 5'-TGCTGTCCCTGTACGCCTCTG-3' (forward primer 1) and 5'-ATGTCCCGCACGATCTCCC-3' (reverse primer 1). The PCR product was 220-bp long. The following gene-specific primers were used to perform semi-quantitative RT-PCR for porcine PBK gene identification and expression profile analysis: 5'- TCTGTGGGAAATGATGAC-3' (forward primer 2) and 5'-ACTTCCAAACAGCCTAAC-3' (reverse primer2). The PCR product was 385-bp in length. The 25 μL reaction system was: 2 μL of cDNA (100-500 ng), 5 pmoles of each oligonucleotide primer (forward primers 1 and 2, reverse primers 1 and 2), 2.5 μL of 2 mmol/L mixed dNTPs, 2.5 μL of 10xTaq DNA polymerase buffer, 2.5 μL of 25 mmol/L MgCl_2_, 1.0 unit of Taq DNA polymerase, with the final addition of sterile water to reach a volume of 25 μL. The PCR program initially started with 94 °C denaturation for 4 min, followed by 25 cycles of 94 °C for 50 s, 55 °C for 50 s, 72 °C for 50 s, ending with a final extentsion at 72 °C for 10 min.

Quantification of the PCR products was carried out by using Glyco Band-Scan software (PROZYME®, San Leandro, California) and the ratio of PBK/beta-actin was calculated using the common EXCEL program. Significant differences in PBK/beta-actin ratios were analyzed by means of the least square method (GLM procedure, SAS version 8.0).

###  5'- and 3'-RACE

5'- and 3'-RACE was undertaken according to instructions in the BD SMART RACE cDNA Amplification Kit (BD science, USA). The Gene-Specific Primers (GSPs) were: 3'-RACE GSP: 5'- AAGGCAGACATATTTGCCTTTGGCC-3', 5'-RACE GSP:5'- AGCAGAAGGGCGATCCTTAGGGTCT -3'.

RACE touchdown PCRs were carried out with 5 cycles at 94 °C for 30 s and 72 °C for 3 min, followed by 5 cycles at 94 °C for 30 s, 70 °C for 30 s and 72 °C for 3 min, and finally with 30 cycles at 94 °C for 30 s, 68 °C for 30 s and 72 °C for 3 min to terminate the reaction. The RACE PCR products were then cloned into PMD18-T vector (TaKaRa, Dalian, China), to be bi-directionally sequenced by the commercial fluorometric method. At least five independent clones were sequenced for each PCR product.

###  Sequence analysis

GenScan software was used for cDNA sequence prediction, and the Conserved Domain Architecture Retrieval Tool of BLAST at the National Center for Biotechnology Information (NCBI) server and ClustalW software for protein prediction and analysis. Phylogenetic tree analysis was carried out with Jalwiew software.

## Results

###  mRNA differential display

**Figure 1:**
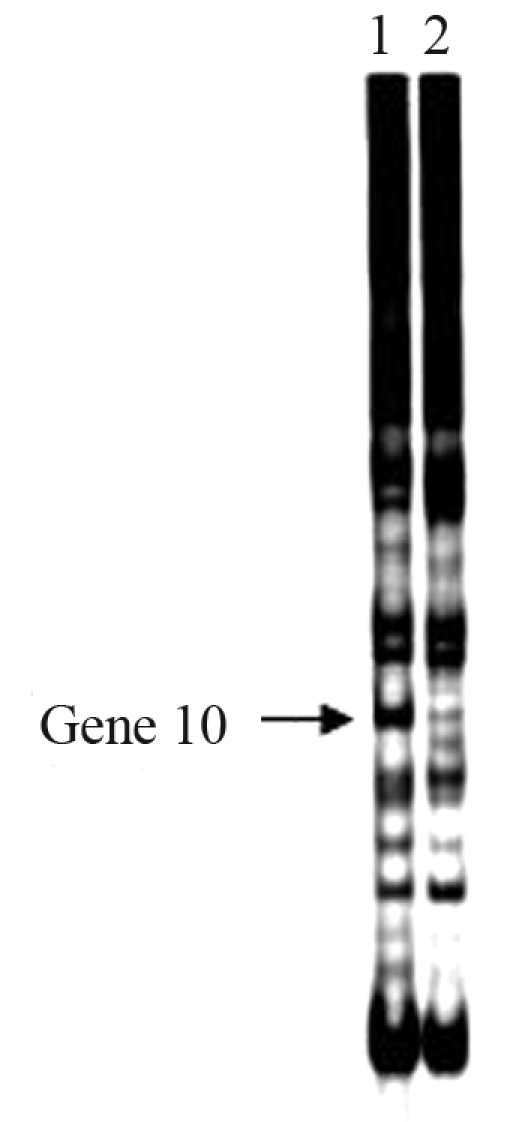
Differential expression analysis of the porcine Gene 10 in longissimus muscle. The arrow indicates the cDNA profile for Gene 10 on an 8% polyacrilamide gel stained with silver nitrate. 1 - Large White; 2 - Meishan.

From mRNA differential display, one gene, denominated Gene 10 and later identified as the PBK gene, was found to be expressed at very low levels in the longissimus muscle of Meishan pigs, whereas it was over-expressed in the longissimus muscle of Large Whites, as shown in Figure 1.

###  Semi-quantitative RT-PCR identification

**Figure 2:**
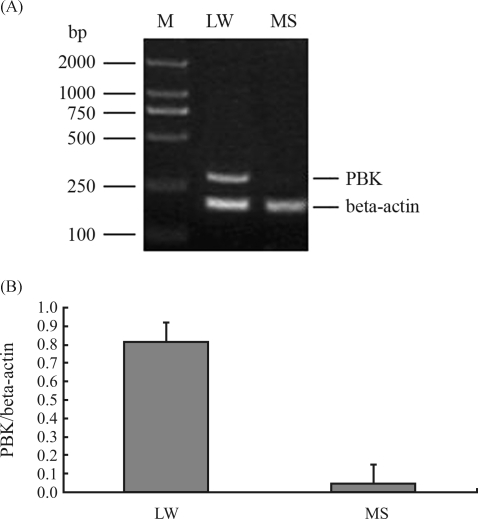
Semi-quantitative RT-PCR identification of Gene 10 (PBK) expression. (A) RT-PCR analysis of Gene 10 (PBK) on a 1% agarose gel stained with ethidium bromide; (B) PBK mRNA expression levels relative to beta-actin; error bars indicate standard deviations (n = 5). M-DL2000 DNA marker; LW – Large White; MS – Meishan.

The differentially expressed gene band was recovered from the gel and used as the template for re-amplification, which was performed with the corresponding oligo (dT) primer and the arbitrary primers used in mRNA differential display. The resultant purified re-amplification PCR product was then cloned into the T-vector and the recombinant plasmid was sequenced. The resulting PCR product was 561 bp. This was in agreement with the result of mRNA differential display. Semi-quantitative RT-PCR was then carried out using these 561-bp fragment specific primers, the results being presented in Fig. 2.

Semi-quantitative RT-PCR results indicated that Gene 10 was over-expressed in the longissimus muscle of Large White pigs and only weakly expressed in the longissimus muscle of Meishans. This also coincided with the results from mRNA differential display.

###  5'- and 3'-RACE

Through 5'-RACE, one PCR product of 1095 bp was obtained. The 3'-RACE product was 1067 bp. These products were then cloned into a T-vector and subsequently sequenced. Taken together, a 1910-bp cDNA complete sequence was finally obtained and this sequence showed no differences between Meishan and Large White pigs.

###  Sequence analysis

**Figure 3:**
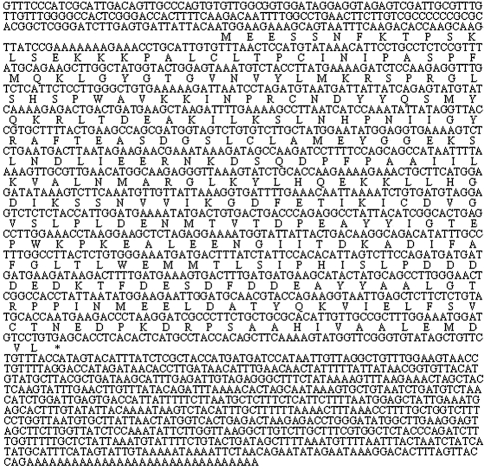
The complete cDNA sequence and encoded amino acids of Gene 10 (GenBank accession number: EU402600). In bold are the ATG start codon and the TGA, stop codon.

The nucleotide sequence analysis using the BLAST revealed that this gene was not related to any known porcine gene. It was subsequently deposited into the GenBank database (Accession number: EU402600). Sequence prediction was carried out using GenScan software. An open reading frame encoding 322 amino acids was found in the 1910-bp cDNA sequence. In the predicted results, exon probability was 0.932 and the poly-A signal was located at 1439 to 1444 bp (consensus: AATAAA). Further BLAST analysis of this protein revealed that it has high sequence identity with a PDZ binding kinase (PBK) of eleven species – dog (accession number: XP_534564; 94%), horse (accession number: XP_001492995; 94%), cattle (accession number: NP_001095648; 92%), human (accession number: BAB55019; 91%), chimpanzee (accession number: XP_520045; 91%), crab-eating macaque (accession number: BAE00991; 89%), rhesus monkey (accession number: XP_001109768; 89%), rat (accession number: NP_001073406; 89%), mouse (accession number: BAB23029; 87%), gray short-tailed opossum (accession number: XP_001364866; 81%) and platypus (accession number: XP_001508951; 83%). This gene was finally denominated GeneID: 100141310. Its complete cDNA sequence and the encoded amino acids are shown in Figure 3.

From the sequencing and structural results heretofore described, this gene can be defined as the porcine PBK gene. Based on the results from the alignment of seven different species of PBK, a phylogenetic tree was constructed as shown in Figure 5. An analysis of this phylogenetic tree revealed that the porcine PBK gene has a closer genetic relationship with the platypus PBK gene than with those of dogs, horses, humans, chimpanzees, crab-eating macaques, rhesus monkeys, rats, mice, gray short-tailed opossums and cattle.

###  Tissue expression profile

Semi-quantitative RT-PCR analysis of tissue expression profiles was carried out using tissue cDNAs of one adult Meishan x Large White pig-cross as the templates. Tissue expression analysis indicated that porcine PBK genes are highly expressed in muscle, moderately expressed in spleen, weekly expressed in heart, liver, lung and kidney, but hardly at all in fat and the small intestine.

## Discussion

PBK encodes a serine/threonine kinase related to the dual-specific mitogen-activated protein kinase kinase (MAPKK) family. From evidence it can be inferred that mitotic phosphorylation is required for its catalytic activity. This mitotic kinase may be involved in the activation of lymphoid cells and support testicular functions, with an implied role in the process of spermatogenesis (Simons-Evelyn *et al.*, 2001; Dougherty *et al.*, 2005; Zhu *et al.*, 2007; Nandi *et al.*, 2007). To data, there is no report on the porcine PBK gene.

From the abovementioned results, we found that the PBK gene was differentially expressed in the longissimus muscle in both Meishan and Large White pigs. Meishan is a fat-type breed, comprised of much more body fat than lean meat or muscle. On the other hand, the Large White is a typical lean-type pig breed, thus presenting the opposite phenotype to that described for the Meishan breed. Regarding body-muscle percentages, the two divergent breeds disclose the trend of Large White-high, Meishan-low. It is very interesting that the expression of the swine PBK gene in the longissimus muscle also follows the same trend, Large White-high, Meishan-low. As we already know, phenotypic variances are mainly determined by gene expression differences. Could it be that porcine PBK gene expression is associated with development or metabolic processes in swine muscle tissue? Based on the previously described functions, PBK is a serine/threonine kinase required for mitosis, which is an important process in cell proliferation and tissue development. All the above evidence implies that PBK protein expression in Large White muscle tissues is higher than in Meishan, thus the former possesses a much higher PBK-associated cell mitosic capacity in these tissues than the latter. Therefore, Large Whites have developed a higher percentage of muscle tissues than Meishans. This deserves to be studied.

**Figure 4:**
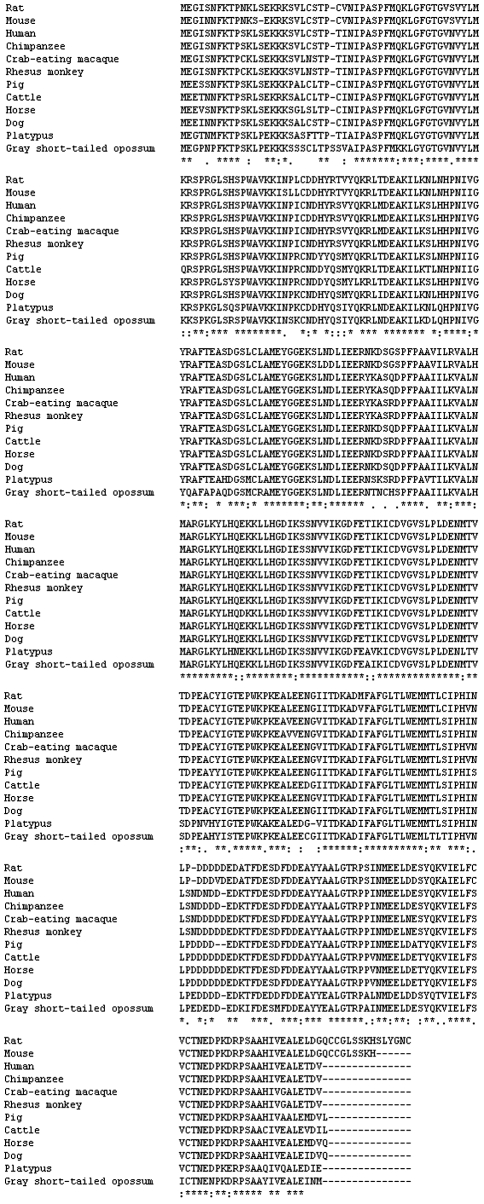
The alignment of the twelve kinds of PBK proteins.

**Figure 5:**
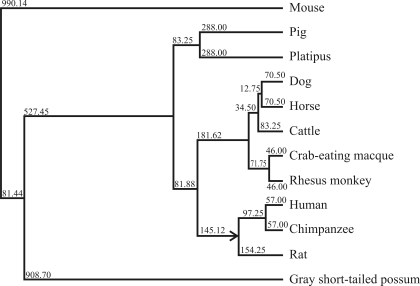
The phylogenetic tree for twelve kinds of PBK genes.

**Figure 6:**
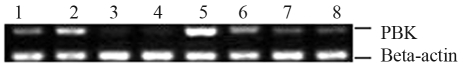
Expression profile of the differentially expressed porcine PBK gene in tissue samples of an adult Meishan x Large White pig-cross. Lane 1, heart; 2, spleen; 3 small intestine; 4, fat; 5, muscle; 6, liver; 7, lung; 8, kidney.

Through tissue expression profile analysis, we also found that the porcine PBK gene was differentially expressed in other tissues. Could this gene also be associated with metabolic processes in these tissues? This is also worthy of study.

In this experiment, we only obtained the cDNA sequence of the porcine PBK gene. Furthermore, we found this gene to be differentially expressed, both in Meishan and Large White pigs themselves, as well as in various tissues. Is it possible for this gene to also be differentially expressed in other pig breeds? If so, and to better understand the function of the porcine PBK gene, further research based on these primary results is required.
